# An investigation into the use of MMCTP to tune accelerator source parameters and testing its clinical application

**DOI:** 10.1120/jacmp.v14i2.3692

**Published:** 2013-03-04

**Authors:** Elaine Conneely, Andrew Alexander, Gabriella Stroian, Jan Seuntjens, Mark J. Foley

**Affiliations:** ^1^ School of Physics National University of Ireland Galway Galway Ireland; ^2^ Medical Physics Unit McGill University, Montreal General Hospital (L5‐113) Montreal Quebec Canada

**Keywords:** Monte Carlo, source optimization, tuning, IMRT

## Abstract

This paper presents an alternative method to tune Monte Carlo electron beam parameters to match measured data using a minimal set of variables in order to reduce the model setup time prior to clinical implementation of the model. Monte Carlo calculations provide the possibility of a powerful treatment planning verification technique. The nonstandardized and nonautomated process of tuning the required accelerator model is one of the reasons for delays in the clinical implementation of Monte Carlo techniques. This work aims to establish and verify an alternative tuning method that can be carried out in a minimal amount of time, allowing it to be easily implemented in a clinical setting by personnel with minimal experience with Monte Carlo methods. This tuned model can then be incorporated into the MMCTP system to allow the system to be used as a second dose calculation check for IMRT plans. The technique proposed was used to establish the primary electron beam parameters for accelerator models for the Varian Clinac 2100 6 MV photon beam using the BEAMnrc Monte Carlo system. The method is intended to provide a clear, direct, and efficient process for tuning an accelerator model using readily available clinical quality assurance data. The tuning provides a refined model, which agrees with measured dose profile curves within 1.5% outside the penumbra or 3 mm in the penumbra, for square fields with sides of 3 cm up to 30 cm. These models can then be employed as the basis for Monte Carlo recalculations of dose distributions, using the MMCTP system, for clinical treatment plans, providing an invaluable assessment tool. This was tested on six IMRT plans and compared to the measurements performed for the pretreatment QA process. These Monte Carlo values for the average dose to the chamber volume agreed with measurements to within 0.6%.

PACS number: 87.55.km

## I. INTRODUCTION

One of the issues with the implementation of Monte Carlo dose calculation methods in a clinical environment is the inconsistent and laborious process of optimizing the linear accelerator source model to be used for accurate simulations. As Monte Carlo simulation methods become more widely accessible in research and clinical applications, it has become a viable option for clinical treatment planning and dose delivery verification.^(^ ^1^ ^)^ The Monte Carlo planning calculations can only be as accurate as the accelerator model being used. While the vendor, under nondisclosure agreement, usually supplies geometric and quantitative specifications for the linear accelerator components and radiation source, the parameters for the electron source (size, energy distribution, and intensity profile) are usually less accurate, and vary from device to device, requiring tuning. Another approach to the problem is to perform separate, more specialized, measurements of parameters determining the characteristics of the source model such as, for example, the electron spot size, but these usually require additional nonroutine experimental work.^(^ ^2^ ^)^

In the work by Pena et al.^(^ ^3^ ^)^ for the Siemens PRIMUS (Siemens AG, Erlangen, Germany) and Varian 2100 CD (Varian Medical Systems, Palo Alto, CA), the initial electron energy is varied in steps of 0.25 MeV over an energy range of 5.5 to 6.5 MeV. For the Siemens KD, Varian Clinac, and Elekta SL25 (Elekta, Stockholm, Sweden), Sheikh‐Bagheri and Rogers^(^ ^4^ ^)^ vary the energy in steps of 0.1 MeV over a range from 5.5 to 6.6 MeV, and Tzedakis et al.^(^ ^5^ ^)^ use increments of 0.2 MeV for the energy tuning from 5.0 to 7.0 MeV for the Philips/Elekta SL75/5. Pena et al.^(^ ^6^ ^)^ in tuning the Siemens PRIMUS recommend using the profiles from wide fields to tune the model, as they show greater sensitivity to changes in the energy and radius of the primary electron beam. Tzedakis et al.^(^ ^5^ ^)^ vary the radius from 0 to 0.40 cm in steps of 0.02 cm, as do Sheikh‐Bagheri and Rogers,^(^ ^4^ ^)^ over a range from 0.01 to 0.19 cm, though Pena and colleagues use 0.05 cm over a range from 0.5 to 4 mm. Sheikh‐Bagheri and Rogers state that they ignored the angular spread of the initial electron beam value (left it at 0°) for their work, as credible divergences of up to 0.5° showed no observable effect. Other studies (such as that by Sawkey and Faddegon^(^ ^2^ ^)^ for the Siemens ONCOR machine) more accurately describe the source parameters using alternative methods based on additional measurements of nonstandard characteristics.

Some authors use wide field profiles and others use output factors, but this work combines these techniques to generate a concise and efficient tuning method which can be implemented in clinical situations by physicists with minimal experience with Monte Carlo techniques.

As differences of up to 10%–20% between treatment planning systems and measurements have been recorded (for example, in lung cases^(^ ^1^ ^)^, a model tuned to 2%–3% accuracy would provide a useful one‐off verification tool for these complex situations when clinical physicists may need some assurance for atypical clinical cases.

This work aims to establish a simple and efficient tuning method that starts from standard clinical measurements and is suitable for clinical implementation, and to verify its efficacy by using it to tune a Varian Clinac accelerator for a 6 MV photon beam to an accuracy of 2%–3%. This model is then used to calculate the dose for 6 IMRT plans and compare the dose values to those obtained from pretreatment IMRT QA measurements to examine the efficacy of the model.

## II. MATERIALS AND METHODS

### A. Monte Carlo calculations

In this work, the electron beam entering the Clinac model was assumed to be mono‐energetic, as work by Sheikh‐Bagheri and Rogers^(^ ^4^ ^)^ showed that, for a symmetric energy distribution, there is only a weak sensitivity (not large enough to be conclusive) of the depth‐dose values at larger depths to the energy spread of the electron beam. The angular spread of electrons resulting from scatter in air from a narrow collimated beam, for most practical applications, can be approximated by a Gaussian radial distribution.^(^ ^3^ ^,^ ^7^ ^,^ ^8^ ^)^ In order for the BEAMnrc^(^ ^9^ ^,^ ^10^ ^)^ code to be used to calculate accurate dose distributions, the accelerator model must first be benchmarked. The initial electron energy and full width half maximum (FWHM) of the radius of the initial electron beam incident on the target was varied to find the percentage depth dose, dose profile curves, and output factors that match the hospital‐measured data, providing output factors that match within 1% of measured output factor values. It was decided that output factors would be the dominant factor in deciding on the tuned parameters, as it would require less simulation time to calculate the output factors accurately using Monte Carlo. Only an accurate dose value in one centrally located voxel is needed for output factor calculations as opposed to accurate dose values in many voxels, and a centrally located voxel at a shallow depth would tend to have a lower uncertainty, anyway.

MMCTP^(^ ^11^ ^)^ is a radiotherapy research platform, which enables comparison and analysis of dose distributions from treatment planning systems and quality assurance measurements with Monte Carlo‐calculated values on a treatment planning system independent platform. MMCTP was used in this work to speed up the tuning process, as it generates the input files and submits the Monte Carlo jobs automatically. It also speeds up the process since it checks the progress of the simulations and submits another job once one has finished. Once an accurate linear accelerator model has been commissioned, the user can save the BEAMnrc input file as a template input file on which the patient‐specific accelerator model is based in further simulations. These files are linked within MMCTP to a specific treatment machine and energy. The DOSXYZnrc^(^ ^12^ ^)^ part of the EGSnrc code is used to calculate the dose scored in a voxelated geometry. This phantom is defined using MMCTP, whether it is based on CT data or just a user‐defined Cartesian phantom.

Directional Bremsstrahlung splitting (DBS) was used with a splitting number of 2000 but no electron splitting was used. The EGSnrc parameters were left at the default values. The PEGS4 data used were the ICRU data with production threshold for secondary electrons and cutoff energies of 189 keV and 10 keV for electrons and photons, respectively.

### B. Measurements

The percentage depth dose (PDD) and dose profile curves were extracted from the 3D dose data using the dose analysis functions of MMCTP. These calculated curves were compared with data obtained from measurements in the Radiotherapy Department at Jewish General Hospital, Montreal. The data from the Jewish General Hospital were acquired in the IBA blue phantom (48 times $48\times 41\;{\text{cm}}^{3}$) using a CC13 ionization chamber. Each IMRT field was measured with an Exradin Farmer‐type chamber model A12 (Standard Imaging, Madison, WI) with a collecting volume of $0.6\;{\text{cm}}^{3}$. The measured Exradin A12 reading was corrected for environment condition (temperature and pressure), polarity, and ion recombination. The polarity $P_{\text{pol}}$ and ion recombination $P_{\text{ion}}$ corrections in each IMRT field and the reference $10\times 10\;{\text{cm}}^{2}$ field were measured using the voltage reversing method and the two‐voltage technique,^(^ ^13^ ^,^ ^14^ ^)^ respectively. The measurements were carried out with the chamber at 8.5 cm depth in a Solid Water (Gammex rmi, Middleton, WI) phantom.

### C. The tuning process

**Figure 1:**
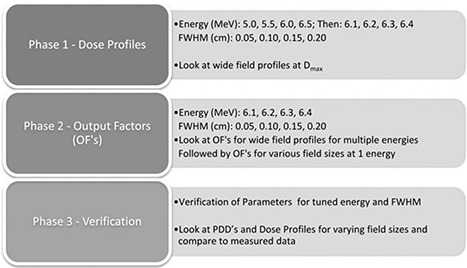
The tuning process as applied to the tuning of the accelerator model.

The tuning process was carried out in three phases. Phase 1 was performed to narrow the energy range estimate by looking at lateral dose profiles for a wide field for a broad range of energy values and FWHM values. Phase 2 used output factors for a wide field to choose the energy that provided the best agreement to measured values, followed by calculating the output factors for a range of field sizes to choose the optimum value for the FWHM of the electron beam. Finally, Phase 3 was carried out to verify the accuracy of the chosen parameters by calculating PDDs and dose profiles for various field sizes and comparing these with measured data of the same. A diagram outlining the tuning process as implemented in this work is shown in Fig. 1.

### D. Clinical application

The model was adapted for use with IMRT plans by using the DYNVMLC component module to model the MLC according to manufacturer's specifications. This model was imported to the MMCTP system, with the initial electron beam parameters chosen from the previous tuning process. The clinical efficacy of the model as verification tool for IMRT plans using MMCTP was tested by using it firstly to recalculate the dose from seven IMRT fields for one plan imported into the MMCTP system from DICOM files exported from the clinical treatment planning system. The doses calculated from this process were compared to measurements carried out with a Farmer chamber located at 8.5 cm depth in a Solid Water phantom, which was modeled using a CT scan of the measurement setup imported into the MMCTP system. Then the composite doses for six IMRT plans were calculated and compared to the doses that were previously recorded during the pretreatment QA process. The dose scoring voxels were set to water of $0.3\times 0.3\times 0.3{\text{cm}}^{3}$. The dose to the chamber was calculated as the average dose to the chamber volume as outlined by the contour on the CT dataset. As the phantom is set to all water, the only effects that voxelating the chamber would have at this resolution would be on the volume (though this should be minimal, as the volume is based on the actual chamber volume) and on the positioning (but again, this should have minimal effect when comparing to measurements as the positioning uncertainty in the measurements would be similar).

## III. RESULTS

The method for tuning that was used in this work starts from standard commissioning data, can easily be automated, and is of reasonable duration. The 6 MV photon model for the Varian linear accelerator was tuned in a number of stages. Initially starting at large field ($30\times 30\;{\text{cm}}^{2}$) dose profiles and PDDs and progressing to examination of output factors for a range of field sizes, focusing particularly on smaller field sizes for finer tuning. Small fields are more sensitive to changes in the FWHM of the radius of the incident electron beam,^(^ ^3^ ^,^ ^15^ ^)^ which allows tuning to be performed more quickly and accurately. It was decided to focus on the larger field sizes initially as these are more sensitive to changes in the mean energy of the initial electron source when looking at profiles at 1.5 cm depth^(^ ^3^ ^,^ ^16^ ^)^ — again improving efficiency and precision of the tuning process being developed.

### A. Phase 1: Energy and FWHM of the incident electron beam

For the initial phase of the tuning process, the energies considered for the 6 MV model were 5.0, 5.5, 6.0, and 6.5 MeV for the electron source. A Gaussian distribution of the radius with a full width half maximum of 0.05, 0.10, 0.15, and 0.20 cm was also used. Large energy increments were used initially to refine the energy range of the primary electron beam, allowing smaller increments to be used subsequently. It was decided not to investigate radii below 0.05 cm, as values below this are unrealistic.^(^ ^17^ ^)^ The water phantom voxels were set to $1\times 1\times 1{\text{cm}}^{3}$ for the measurement of the dose profiles at 1.5 cm depth. This somewhat large voxel size was employed to keep simulation time to a minimum so as to perform quick simulations to narrow the estimation of the energy range. This was necessary because the literature shows values for the energy ranging from 5.7 MeV to 6.3 MeV,^(^ ^16^ ^)^ so these approximate simulations were carried out to narrow the range. All doses have been normalized on the central axis and these preselective calculations were run at a low number of histories (3 to 7 million particles incident on the target, depending on energy) to give a narrower estimation of the source energy in the least amount of time possible. These simulations had an average uncertainty of 1.3%. The results were evaluated using a root‐mean‐square difference value (RMSD). The RMSD value was calculated for the dose profile at 1.5 cm depth (depth of maximum dose for the 6 MV beams for a $10\times 10\;{\text{cm}}^{2}$ field) for each set of initial electron source parameters. The RMSD was calculated from x=−15cm to +15 cm for the $30\times 30\;{\text{cm}}^{2}$ field.

**Figure 2:**
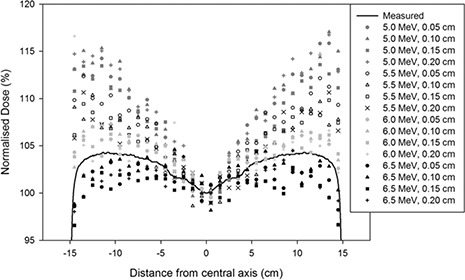
Dose profiles obtained at 1.5 cm depth for a $30\times 30\;{\text{cm}}^{2}$ field from the initial tuning process.

After this initial tuning process, it was decided from the RMSD values that the best value for the initial electron energy would be found somewhere in the region between 6 MeV and 6.5 MeV for the accelerator. This is confirmed by the dose profiles (Fig. 2), as the measurements (continuous black line) can be seen to lie between the 6 MeV (light grey points) and 6.5 MeV (black points) values. So it was decided to continue the tuning process using a reduced step size of 0.1 MeV between 6 and 6.5 MeV, while once again using the same values for the full width half maximum of the radius of the incident electron beam, as the RMSD values did not point to any obvious trend in this. A larger number of histories were used at this stage (8 to 11 million histories striking the target, again depending on energy) as the simulations needed to be more precise to differentiate which energy provided a better match with measured data.

### B. Phase 2: Output factors

**Figure 3:**
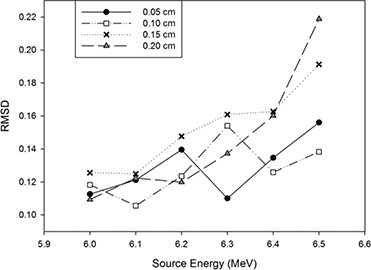
Dose profile RMSD values as a function of energy for the data obtained running simulations with energies of 6.0, 6.1, 6.2, 6.3, 6.4, and 6.5 MeV and FWHM of 0.05, 0.10, 0.15, and 0.20 cm for the incident electron beam.

After the first phase of the tuning process, looking at the RMSD values obtained for the PDDs and dose profiles from Phase 1 of the tuning, it was still not possible to establish which values for energy and radius produced the best match to the measured data. Examination of the plots of the RMSD values deduced from the dose profiles (Fig. 3) show no clear trend in the effects of adjusting the energy or radius of the incident electron beam in Phase 1 of the tuning process. This was due to the differences between the graphs obtained being within the uncertainty of the Monte Carlo calculations. In order to get a lower uncertainty and hence decide upon the optimum values for the initial electron beam, the simulations would become very long and the tuning process unacceptably time‐consuming for clinical implementation (Table 1). Output factors for the $30\times 30\;{\text{cm}}^{2}$ field were used to further finetune the energy as output factors are more sensitive to changes in the primary beam parameters^(^ ^3^ ^)^ and, as a result, require less computation time than extended simulations and profile comparisons. The output factor was calculated from the dose scored in a $0.5\times 0.5\times 0.5\;{\text{cm}}^{3}$ voxel on the central axis at 5 cm depth for the Varian Clinac, as this was the depth the output factors were measured at in the clinic.

**Table 1:** Average computation hours on a 300 core cluster with a clock frequency of 2200 MHz.

*Phase*	*Average Computation Time per Calculation (hrs)*	*Number of Calculations for this Phase*	*Total Computation Hours for this Phase (hrs)*
5.0, 5.5, 6.0, & 6.5 MeV	8.4	16	134.4
6.1, 6.2, 6.3, & 6.4 MeV	25.6	16	409.4
$40\times 40\;{\text{cm}}^{2}$	22.6	12	271.2
$10\times 10\;{\text{cm}}^{2}$	24.3	12	291.6
$5\;{\text{cm}}^{2},\;4\;{\text{cm}}^{2}\;\&\;3\;{\text{cm}}^{2}$	21.9	12	262.8
All fields	38.4	4	153.6

**Figure 4:**
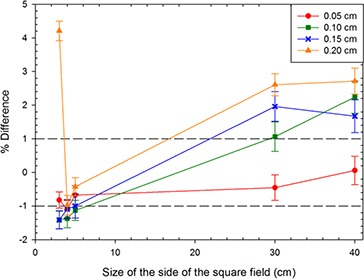
Plot of difference between measured and calculated output factors with the 1% difference lines drawn shown as dotted lines (this was decided as the tolerance for the accuracy of the output factors). The error bars show the percent uncertainty in the Monte Carlo‐calculated dose values from which the output factor was calculated.

For the Varian model, it was determined from these initial output factor investigations (using the simulation results from Phase 1 for the $30\times 30\;{\text{cm}}^{2}$ output factor values) that the 6.3 MeV beam gave the closest and most consistent results when compared with the measured output factor for this field at all radial distributions of the incident electron beam. Subsequently, this allowed the output factors for square fields of different sizes to be calculated, at one energy, of 6.3 MeV. The output factors for square fields of $40\times 40\;{\text{cm}}^{2}$, $30\times 30\;{\text{cm}}^{2}$, $5\times 5\;{\text{cm}}^{2}$, $4\times 4\;{\text{cm}}^{2}$, and $3\times 3\;{\text{cm}}^{2}$ were considered. The inclusion of smaller field sizes allowed for a more accurate determination of the FWHM of the beam radius^(^ ^3^ ^)^ as these are the most sensitive to changes in the spatial distribution, while larger fields are more sensitive to energy variations. From this it was determined that the optimum value for the Gaussian distribution of the radius was 0.05 cm, because this radius value provided output factor values that matched measurements within 1% for all the field sizes investigated (Fig. 4). This had been decided as the desired agreement between calculated and measured central axis output factors.

### C. Phase 3: Verification of the tuning results

**Figure 5:**
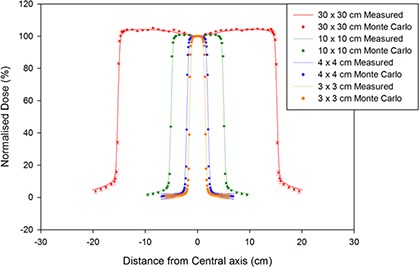
The dose profile comparisons for the tuned Varian model compared with measurements for square fields of side 30 cm, 10 cm, 4 cm, and 3 cm.

**Figure 6:**
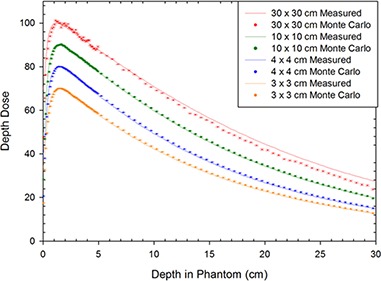
The depth‐dose comparisons for the tuned Varian model compared with measurements for square fields of side 30 cm, 10 cm, 4 cm, and 3 cm.

**Figure 7:**
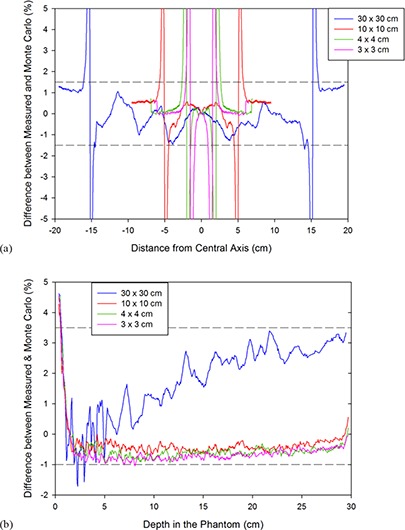
The dose profile differences (a) and PDD differences (b) for the tuned Varian model between Monte Carlo values and measurements for square fields of side 30 cm, 10 cm, 4 cm, and 3 cm.

Finally, once the best values had been decided upon, the dose profiles at 1.5 cm depth and the depth dose curves for different field sizes were simulated to a low uncertainty (average uncertainty of 0.8%) using the tuned primary electron beam energy and FWHM for different field sizes, and compared with measured data to verify the accuracy of the chosen parameters. For the dose profiles, the voxel sizes were adjusted with field size in order to obtain a more accurate shape of the penumbra, while using larger voxels outside this region of the dose profiles to keep the simulation time to a minimum (Table 2). The tuned dose profiles for the Varian model can be seen in Fig. 5, while the PDDs as calculated using the tuned model are visible in Fig. 6 and the difference plots for the dose profiles and PDDs are in Figs. 7(a) and (b), respectively.

**Table 2:** Voxel sizes used for the dose profiles at 1.5 cm depth for the verification of the tuning results simulations.

*Field Size*	*1st Group*	*2nd Group*	*3rd Group*	*4th Group*	*5th Group*
$30\times 30\;{\text{cm}}^{2}$	1 cm×4	0.2 cm×10	1 cm×28	0.2 cm×10	1 cm×4
$10\times 10\;{\text{cm}}^{2}$	1 cm×4	0.2 cm×10	1 cm×8	0.2 cm×10	1 cm×4
$4\times 4\;{\text{cm}}^{2}$	0.3 cm×14	0.1 cm×15	0.2 cm×13	0.1 cm×15	0.3 cm×14
$3\times 3\;{\text{cm}}^{2}$	0.3 cm×13	0.1 cm×14	0.3 cm×6	0.1 cm×14	0.3 cm×13

Every effort has been made in this work to tune the models in the minimum amount of simulation time possible (see Table 1), while maintaining a clinically reasonable level of accuracy (2%–3%) in the resulting calculated doses and keeping the complexity of the tuning process to a minimum. However, variance reduction techniques (VRTs) were not examined in this work; focus was placed on devising a concise tuning process and testing its application on IMRT fields. VRTs were not required, as the use of a computing cluster meant that simulations could be run in parallel. For those applying this technique without the use of such computational facilities, the use of VRTs can further speed up the tuning process and the techniques are covered extensively in the literature.^(^ ^10^ ^,^ ^18^ ^,^ ^19^ ^)^ All the measured values required for this tuning method are routine QA measurements and are generally readily available in the clinic. Consequently, this method does not require the laborious process of obtaining specialized individual measurements.

It was hoped that the inclusion of small fields in the tuning process would allow the model to be used for the simulation of IMRT fields. This application was tested by incorporating the model into the MMCTP system to recalculate the dose for an IMRT patient plan to a chamber in a Solid Water phantom. This dose was compared to the dose measured, under the same setup as simulated, for the QA process. This allows the MMCTP system to provide a second dose calculation check for IMRT plans. The results (Table 3) of the beam‐by‐beam simulations were good (within 3%), apart from the final beam (5.7% difference). The difference between the measured dose and that from the treatment planning system was also largest for this beam, indicating that there may have been an issue with this measurement for this field. Boyer et al.^(^ ^20^ ^)^ estimate a 3% uncertainty in IMRT QA measurements. Due to the complicated dose intensity distributions and small treatment fields associated with IMRT deliveries, significant measurement uncertainty may arise due to setup errors in positioning the ion chamber.

**Table 3:** Results of IMRT beam‐by‐beam dose recalculation. All dose values are in cGy. Percentage differences are given between measured values and calculated values.

*Beam*	*MC Uncert. (%)*	*Measured*	*DOSXYZ (% Diff)*	*TPS (% Diff)*
1	0.79	24.86	24.57 (−1.17)	25.2 (1.37)
2	0.81	22.13	21.66 (−2.12)	22.29 (0.72)
3	0.79	27.63	26.85 (−2.82)	27.25 (−1.38)
4	0.72	32.3	31.32 (−3.03)	31.83 (−1.46)
5	0.80	24.61	24.37 (−0.98)	25.13 (2.11)
6	0.81	20.19	19.78 (−2.03)	20.07 (−0.59)
7	0.90	15.52	14.64 (−5.67)	15.1 (−2.71)

The composite IMRT simulations were done for six patients with 7–9 dynamic beams, with all beams directly intersecting the chamber. DOSXYZnrc results of average dose to the chamber agree with measurements to within 0.6%. The treatment planning system dose values agree with measurements to within 1.8% for the same plans (Table 4).

**Table 4:** Results of IMRT plan QA dose recalculation. All dose values are in cGy. Percentage differences are given between measured values and calculated values.

*Plan*	*No. of Beams*	*Measured*	*DOSXYZ (% Diff)*	*TPS (% Diff)*
1	7	164.60	164.40 (−0.14)	166.9 (1.28)
2	7	153.50	153.1 (−0.25)	155.7 (1.43)
3	9	354.00	354.8 (0.21)	353.4 (−0.10)
4	7	132.00	133.1 (0.57)	134.7 (1.74)
5	7	205.30	204.9 (−0.20)	206.0 (0.39)
6	7	259.90	258.7 (−0.47)	257.1 (−1.00)

## IV. DISCUSSION

For the Varian Clinac, the dose profile simulations and initial output factor calculations indicated that the 6.3 MeV electron energy provided the best fit over the range of FWHM values to the measured values. Investigation of the output factors obtained for different square fields ($40\times 40,30\times 30,5\times 5,4\times 4,\;\text{and}\;3\times 3\;{\text{cm}}^{2}$) at the different radial distributions determined a value for the beam radius of 0.05 cm. This produced a match within 1% of the measured values for the output factors at all field sizes. Once the values were determined for the initial electron beam parameters, the PDDs and dose profiles were simulated for different field sizes and compared to measurements to verify the results.

For the Varian model, the PDDs all agree within 1% after 1.5 cm depth, apart from the $30\times 30\;{\text{cm}}^{2}$ which is within 3.5% after 1.5 cm, though Pena et al.^(^ ^3^ ^)^ suggest the large differences for the $30\times 30\;{\text{cm}}^{2}$ field are not very clinically relevant. The Varian model dose profiles agree within 1% outside of the penumbra for the smaller field sizes and within 1.5% outside of the penumbra for the $30\times 30\;{\text{cm}}^{2}$ field, and within the penumbra they agreed within 3 mm, though this is considered quite reasonable as the detector used for the measurements had a diameter of 5 mm. Pena and colleagues also had worse agreement with larger fields for the Varian 2100 6 MV Clinac model. Chibani et al.^(^ ^16^ ^)^ suggest that this could be due to inaccuracies in the actual geometry of the Clinac model as this can only be modeled as accurately as the information the vendor provides. These verification simulations were calculated with an average uncertainty of up to 0.8%.

The value for the primary electron beam energy (6.3 MeV) is in keeping with the values obtained by other authors^(^ ^3^ ^,^ ^4^ ^,^ ^7^ ^)^ generally between 5.7 and 6.5 MeV. The beam radius value obtained (0.05 cm FWHM) is comparatively smaller than the values reported by Pena et al.,^(^ ^3^ ^)^ Sheikh‐Bagheri and Rogers,^(^ ^4^ ^)^ and Keall et al.,^(^ ^7^ ^)^ where it has been shown to be between 0.1 cm and 0.2 cm. However, this value is plausible, as measurements of focal spot sizes^(^ ^17^ ^)^ have shown that a FWHM of 0.05 cm radius is possible as a FWHM of between 0.12 and 0.14 cm diameter for the Varian Clinac 2100 has been recorded. Sham et al.^(^ ^15^ ^)^ also obtain a FWHM of 0.15 cm diameter for a 6 MV beam for a Varian Clinac. In addition, our values are only correct to within 0.05 cm, as this was considered sensitive enough for the purposes of this investigation.

A more rigorous and time‐consuming tuning process could provide a model tuned to 1% as implemented in the Pena study or 1.5% in the Sheikh‐Bagheri study. However, for the purposes of this investigation an accuracy of 2%–3% was deemed adequate.

## V. CONCLUSIONS

An efficient tuning method has been implemented using the MMCTP system and the BEAMnrc Monte Carlo code. The aim was to establish a model that agreed to measured data to within 2%–3% so that it could be used as a verification tool for nonstandard clinical treatment plans. The final model fit this requirement, apart from the $30\times 30\;{\text{cm}}^{2}$ PDD at greater depths (≈20 cm depth or greater). This model was successfully used in recalculating the dose to the chamber for six IMRT plans, further verifying the efficacy of the model. This work shows that the goal of establishing a convenient and minimally time‐intensive method for all linac models' tuning suitable for clinical implementation has been achieved.
